# The Prophylaxis of Second Dentition

**Published:** 1912-06-15

**Authors:** N. S. Hoff


### THE PROPHYLAXIS OF SECOND DENTITION.

BY N. S. HOFF, D.D.S.

*Incisor Malpositions.* In a former paper we gave the prophylaxis of the eruption of the first permanent molars and we need not here repeat that consideration. The next of the permanent dentures to emerge from the gums are the incisors. What are the conditions which influence these teeth to develop and erupt in abnormal positions and how can helpful treatment be applied?

The lower incisors are the first to erupt ordinarily and they usually emerge to the lingual of the deciduous incisors, unless the roots of the deciduous teeth absorb normally, in which case the permanent tooth simply replaces the deciduous tooth in its socket. The more common and troublesome malposition is a torsal emergence caused by the flattened and broad shape of the incisal end of the crown of the permanent tooth which being broader than the root of the deciduous tooth causes the whole tooth to rotate on its axis that it may crowd into the limited space which at this time is the easiest thing for it to do as its root is not yet completely calcified and the peridental structures not completely formed. It will be evident therefore that the treatment needed to prevent both of these malpositions is to provide sufficient space before the permanent teeth start to erupt. This can best be accomplished by placing a soft german silver wire between the lingual surfaces of the cuspids. It may be anchored by drilling small pits in the enamel and pointing the ends of the wire and then springing it into place. By pinching this wire every few days with a pair of pliers which will slightly lengthen it the needed space may be secured in a few weeks for the emergence of all the incisors in proper alignment. If the four lower incisors can be brought into normal postion in this way the temporary cuspids will also be forced into a larger arc and so assist materially in preventing the permanent cuspids from crowding the incisors out of alignment when they begin to erupt. The incisors begin to emerge at the sixth or seventh year. It will be safe prophylaxis to begin preparation for them as soon as the lower first molar shows itself.

It may not always be easy to determine whether any preventive treatment is needed. If the deciduous teeth are small and closely set it is highly probable that treatment will be needed, but if the teeth are well spaced and the arch reasonably well developed no treatment need be made until it is evident the teeth will emerge into wrong positions. A serious mistake is often made by parents and dentists also, in deferring any treatment until the permanent teeth are fully erupted. The usual result is a complicated treatment involving the entire arch and usually both arches. If the interference can be made at the proper time we not only provide space for the immediate needs of the incisors but the stimulus given the incisal region of the arch extends to the whole arch and we have a corresponding extra development for the teeth coming on later.

*Retruding Chin.* In some cases there is an arrest of development in the mandible and there is a noticeable deficiency in the chin development. If this is embryonic it can not usually be remedied. In some cases the alveolus also fails to develop, and when the teeth do erupt they are both crowded and malposed, even the deciduous teeth show the deformity. In some cases the incisal portion of the teeth tip forward and protrude the lip. If the child at an early age can be induced to eat hard food and masticate thoroughly the massaging effect on the trophic function will do more than anything else to cause a development of the mandible, which is highly desirable. The trouble usually is that these cases are not seen by the student of prevention until completely formed and then it becomes wholly an orthodontic procedure. By an early adjustment of orthodontic arches much may be done to stimulate interstitial bone growth. This procedure must be intelligently made or it will only result in tipping the teeth and forcing them forward in the arch producing a facial abnormality almost as objectionable as the malocclusion of the teeth.

It has been claimed that this lack of development of the mandible is hereditary and embryonic and consequently not preventable. From personal observation and experience in treatment I believe that it is more often accidental, and due to interference with the nutritive forces, by such causes as diseased tonsils and adenoid growths. We have seen many cases make good recovery after treatment involving the removal of these causes. Of course such treatment would be ineffective where serious degeneracy of the nervous system existed, as in mental deficiencies or imbecility. The common notion that a retruding chin is indicative of defective mental and moral character is wholly fictitious, as in the large majority of cases it is purely accidental and should have been corrected when the jaws and teeth were in the developing stages. It is undoubtedly true that enlarged tonsils and adenoid growths interfere with mental development as they do with the jaws and teeth, because of their almost direct, or at least their intimate reflex associations. There is no time so favorable for the treatment of these conditions as the time when the second teeth are beginning to erupt and no oral prophylaxis which ignores the throat and post nasal region at this time will be thorough and efficient.

*The Upper Incisors.* The eruption of the upper incisors follows very soon after the lowers, and much the same influences predispose their relations. These teeth usually emerge to the labial of the deciduous teeth; although the laterals if delayed may be forced to the lingual by the cuspids. Because of the anatomical form of the central roots they are easily rotated and there is a strong tendency if the crowns are broad and flat and the space contracted for them to rotate mesially. The preventive treatment here is the same as for the lower; namely widen the deciduous arches at the cuspid regions. If this is done promptly there will be no rotation of the incisors and should there be a labial or lingual emergence it can be corrected easily.

One of the most troublesome malpositions of these teeth is a labial protrusion. This may be the result of arrested development in the facial and maxillary bones producing a contracted arch and a narrow vault, which forces the incisor teeth forward into an angular or narrower arc with a considerable protrusion and overbite. In other cases there is an excessive development of bone in the molar regions and a consequent open bite which prevents the upper and lower incisors from occluding. This hyperplastic condition is somewhat obscure in its etiology. It is generally associated with obstruction in the nasal passages compelling mouth breathing. Possibly the pharyngeal irritation is so great in the formative period as to cause the hyperplasia from the near contact of the structures implicated. I do not recall ever having seen a marked case in which there was not present tonsilar affections of both the throat and post nasal tissues.

The preventive treatment is thorough removal of all diseased adenoid tissue before the time for the emergence of the permanent teeth. If this is not done the case becomes one of repair, or orthodontia. These conditions are readily recognizable at a sufficiently early date to enable the removal of the cause of the deformity before it does irrespairable harm. These operations can be made from the third to fifth year with great benefit. Physicians do not realize the importance of the early and thorough treatment of these cases and parents are prone to delay until the child is of an operable age. It should be the dentists duty to point out the harm likely to result and counsel such treatment as may be indicated.

*Bicuspid Malpositions.* In the upper jaw the most common malposition is the lingual curving from the normal, producing what Talbott has called the saddle arch. This displacement is due to mouth breathing which stretches the buccal muscles abnormally making constant pressure on the buccal sides of the arch with a strong force which is not counter balanced by the tongue and occlusal forces of the teeth in antagonism. The lack of development in the nasal and facial bones also contribute to this deformity. In most cases the teeth are not only lingually placed, but because of their eruption into crowded arches they are frequently more or less in torsal positions. This may also be the result of too long retention of the deciduous molars, whose roots fail to absorb because of their atrophic environment.

The remedy is the expansion of the deciduous arch at the time for the normal loss of the deciduous molars and their replacement by the bicuspids. If there is delayed development because of disease of the accessory organs of respiration this should of course have immediate attention. Diseased adenoids and other unfavorable conditions should be removed and then the expanding forces applied in such manner as to produce slight but sufficient pressure to cause normal or slightly excessive stimulation of soft tissue and bone growth. This force may be applied with advantage to the deciduous molars even after their root absorption has begun. It will be found that even this slight pressure may be kept up until the deciduous molars are so loose that they can easily be removed. Care of course must be taken to not apply the force excessively as premature loosening of the molars may occur. If the case does not present for treatment before the deciduous molars have been lost and the bicuspids have begun to erupt into abnormal relations the problem of treatment is difficult and generally no direct treatment can be applied to the bicuspids as their crowns have not sufficiently erupted to provide anchorage for appliances. In such cases the expansive force must be applied to the molars to the extent of encouraging development in the arches only, and not to produce widening mechanically. If the deciduous cuspids remain which is usually the case they can also be included with the permanent first molars by ligaturing them to the arch.

The torsal position of the bicuspids can not be corrected until they have erupted sufficiently to enable them to be banded and attached to the arches.

If the deciduous teeth occlude normally the most effective prevention is to induce the child to use them even to excess, if necessary, to produce bone growth through the normal stimulus of masticating the harder foods. Teach the child to use the teeth properly, that is to eat such foods as require considerable grinding in order that they may be comfortably swallowed. Nut meats, twice baked bread, meats, &c., which can not be comfortably swallowed until well triturated and insalivated should be the larger part of a childs diet, rather than soups, puddings and other predigested paps. There is no portion of the arches in which normal development is oftener required than in the bicuspid and first molar region. The cusps of these teeth should occlude in exact normal relations as the effectiveness of future mastication is wholly dependent on the correct occlusion of these teeth, it is by them that the heavy mastication is to be done. Therefore they should be in perfect alignment and without torsal position. Owing to the conical form of the roots the lower bicuspids are more likely to be rotated than the upper.

*The Cuspids.* They are the last to erupt and complete the arch, they have been called the keystones. They do not appear until the bicuspids are well erupted. Usually they appear to the labial,—occasionally the uppers appear to the lingual of the arches. They have the longest roots and develop with so much power that they spread the arches and make room for themselves, where none seems to exist. Very often they will crowd the already erupted teeth out of position that they may take their normal place. While these teeth often seem prominent and out of alignment it will usually be found that they are more nearly in normal position than the other teeth. The best preventive treatment is to preserve the deciduous cuspids as long as possible and at the same time secure as much space as possible for their coming. The cuspids and bicuspids of the upper sometimes erupt into buccal positions and must be forced into alignment. The lower bicuspids erupt most frequently into lingual occlusion and seldom into buccal positions.

Normal postion of the permanent teeth can be most easily secured by preventive treatment for the usual destructive agencies which cause premature decay of the deciduous teeth. Constant care in the prevention of caries and the inculcation of habits of oral cleanliness, with instructions as to vigorous use of the teeth on the harder foods will do more than all else to secure normal development and typically normal occlusion of the permanent teeth. Operative or corrective interference should not be the rule or the common practice as at the present time.

A typically developed set of permanent teeth in normal alignment and occlusion ought never to suffer from caries or other oral diseases if kept in use and under careful personal and professional surveillance.

